# Supplementary material for: Effect of agricultural extension on technology adoption by Palestinian farmers under Israeli occupation in the West Bank
Source: PLoS One. 2023 Nov 8;18(11):e0294023. doi: 10.1371/journal.pone.0294023 (PMC10631675; doi:10.1371/journal.pone.0294023)
Supplement: Attachment — Submitted filename: Hilmi S. Salems Comments_29 August 2023.pdf [file pone.0294023.s003.pdf]

New comments on the improved manuscript: PONE-D-22-25946R1
Dated and Submitted on: 29 August 2023

Dear Respected Editor-in-Chief:
Dear Respected Authors:

First, it is my great pleasure to review the improved version of the Manuscript (Ms) re-submitted by scholar colleagues from Japanese universities. I appreciate their efforts.

Commenting on the "improved" manuscript re-submitted by the authors, please find below my recent (new-old) comments and kindly feel free to send this letter, with the comments below, to the authors, considering the fact that the authors have NOT responded positively to some of my comments given in the fist review, while they responded well on some other comments that I have made.

Accordingly, most of my comments below are with reference to my comments in the first review.

My final decision, based on the comments below, I support publishing the Ms in your Journal "Plos One", if the authors consider the comments mentioned below. However, the final decision is the Journal's Editorial team.

1) Abstract: "Occupied Palestinian Territories (OPT) as an urgent issue."

2) Abstract and throughout the paper: "by the Israeli Occupation Authorities …"

3) Throughout the paper, the authors still use the term "West Bank Barrier," though I asked them to replace it with the term "Israeli Segregation Wall," as widely used by local and international researchers and documented in a plenty of literature. It is not a small barrier or fence between good neighbors but a giant monstrous wall intended to confiscate huge Palestinian lands and water resources behind it, and to significantly and negatively impact the life of the entire Palestinian population in the occupied West Bank, including occupied East Jerusalem. Its length has reached, so far, about 800 km surrounding the occupied West Bank. Please see this reference of mine, in which you find some description of the Israeli Segregation Wall built illegally on Palestinian lands of the occupied West Bank. Please feel free to cite and reference it in your current Ms. and other future works:

   Salem, H.S. (2011). Social, environmental and security impacts of climate change on the Eastern Mediterranean. In: Brauch, H.G., Spring, Ú.O., Mesjasz, C., Grin, J., Kameri-Mbote, P., Chourou, B., Dunay, P., Birkmann, J. (Eds.): Coping with Global Environmental Change, Disasters and Security – Threats, Challenges, Vulnerabilities and Risks. The Institute for Environment and Human Security, United Nations University (UNU-EHS). Hexagon Series on Human and Environmental Security and Peace, Berlin – Heidelberg – New York: Springer-Verlag. PP: 421–445. 1815pp. Available at:
   https://link.springer.com/chapter/10.1007/978-3-642-17776-7_23
   https://www.researchgate.net/publication/299562984_Social_Environmental_and_Security_Impacts_of_Climate_Change_of_the_Eastern_Mediterranean

4) Abstract and in other locations: Palestinian Authorit~~ies~~ Authority
5) Abstract: Using the data of the Agricultural Census 2010, The authors did not explain enough why they used this relatively old data!!!!

6) Under Introduction: before and after the 2014 ~~Gaza conflict~~ Israeli war on the Gaza Strip …

7) Page 6: The Israeli Authorities have restricted its use of water resources in much of the West Bank, leading to chronic irrigation water shortages[7]. Please consider adding here (in addition to the 7th reference) this up-to-date (March 2023) reference which was also recommended in the first review, and also the following one which has been published just today. It is belied that both reference are relevant to the Ms.:

   Salem, H.S. (2023). Potential Solutions for the Water Conflict between Palestinians and Israelis. A Book Chapter (PP: 123–185) In: Hussein A. Amery (Ed.): "Enhancing Water Security in the Middle East". MENA Water Security Task Force, Al-Sharq Strategic Research, Al-Sharq Forum, Istanbul, Turkey; and Colorado School of Mines, Denver, CO, USA. Published on 20 March 2023.
   https://research.sharqforum.org/mena-water-security-task-force/
   https://www.researchgate.net/publication/367190828_Chapter_4_Potential_Solutions_for_the_Water_Conflict_Between_Palestinians_and_Israelis

   Salem, H.S. (2023). Socioeconomic, environmental, and health impacts of reusing treated wastewater in agriculture in some Arab countries, including occupied Palestine, in view of climate change. Natural Resources Conservation and Research, Volume 6, Issue 2. 22 Pages. "A special Issue: Climate-Adaptive Approaches to Risk Monitoring and Sustainable Water Resources Management." (Published online: 29 August 2023), EnPress Publisher, El Monte, CA, USA. DOI:
   http://dx.doi.org/10.24294/nrcr.v6i2.2229
   https://systems.enpress-publisher.com/index.php/NRCR/article/view/2229
   https://www.researchgate.net/publication/372317053_Socioeconomic_environmental_and_health_impacts_of_reusing_treated_wastewater_in_agriculture_in_some_Arab_countries_including_occupied_Palestine_in_view_of_climate_change

8) Page 18: (New Israel Shekel) ….

9) Under "Conclusions": However, the effects of the conflict are diverse and devastating on Palestinian farmers in the Occupied Palestinian Territories (West Bank and Gaza Strip). They, however, ~~and~~ cannot be fully explained by the variables used in this study. Therefore, it is still necessary to find and use variables that more accurately represent the impact of the conflict.

10) In the West Bank, the Israeli Occupation Authorities ….

11) The Gaza Strip could not be included in the same model in this study because it has no Israeli settlements. Yes, but the Gaza Strip is totally besieged and blockaded by the Israeli Occupation Authorities since 2006. Please mention this note in your Ms. and support it with references.

12) References: 32. Salem, H.S. 2019. Agriculture Status …..

13) References: 48. Salem, H.S. 2019. No sustainable development ….

I appreciate your attention to this matter.

Kind regards. --- Hilmi

Prof. Dr. Hilmi S. Salem
