# Supplementary material for: Effect of agricultural extension on technology adoption by Palestinian farmers under Israeli occupation in the West Bank
Source: PLoS One. 2023 Nov 8;18(11):e0294023. doi: 10.1371/journal.pone.0294023 (PMC10631675; doi:10.1371/journal.pone.0294023)
Supplement: Attachment — Submitted filename: Response to Reviewers PLOS ONE Nakamura 230707.docx [file pone.0294023.s002.docx]

**Response to reviewers**

**Reviewer #1**

| No. | Comments | Revision results and explanation |
| --- | --- | --- |
| 1 | Overall the article is well written and well organized, however the article has some minor typos. | All the typos in the manuscript were corrected. The manuscript was checked by a native English speaker. |
| 2 | The abstract should follow the following outline: a reminder of the fundamental objective, estimation methods and data, main results, and recommendations. | The abstract was revised according to the recommended structure. Fundamental objective was added at the beginning. |
| 3 | In the presentation of the methodology, the author should present the theoretical model, drawing on the models used in the literature review. | The following explanation was added:  <Results-Estimation of propensity scores>  "In this study, the propensity scores represent determinants to receive agricultural extension for agricultural holdings. The propensity scores were calculated from multiple variables that should influence determinants to receive agricultural extension and other control variables. Those variables selected for the calculation were influence of the conflicts, characteristics of agricultural holdings, lands and agricultural products, and governorates based on previous studies [22] [31] [34] [35] [36]. The theoretical model for the propensity score calculation is shown in Fig 1."  <Reference>  22. Ozaki R, Sakurai T. The Adoption of Upland Rice by Lowland Rice Farmers and Its Impacts on Their Food Security and Welfare in Madagascar. Japanese Journal of Agricultural Economics. 2020;22:106-11. https://doi.org/10.18480/jjae.22.0_106Ramahaimandimby Z, Sakurai T. 2021. Vegetable Production and Its Impact on Smallholder Farmers’ Livelihoods: The Case of the Central Highlands of Madagascar. Japanese Journal of Agricultural Economics. Mar 31,;23:125-30. doi:10.18480/jjae.23.0_125  31. Ramahaimandimby Z, Sakurai T. 2021. Vegetable Production and Its Impact on Smallholder Farmers’ Livelihoods: The Case of the Central Highlands of Madagascar. Japanese Journal of Agricultural Economics. Mar 31,;23:125-30. https://doi:10.18480/jjae.23.0_125  34. Edge, B., Velandia, M., Lambert, D. M., Roberts, R. K., Larson, J. A., English, B. C., Boyer, C., Rejesus, R., & Mishra, A. 2017. Changes in the Use of Precision Farming Information Sources Among Cotton Farmers and Implications for Extension. The Journal of Extension, 55(2), Article 16. https://tigerprints.clemson.edu/joe/vol55/iss2/16  35. Jensen, K. L., English, B. C., & Menard, R. 2009. Livestock Farmers' Use of Animal or Herd Health Information Sources. The Journal of Extension, 47(1), Article 6. https://tigerprints.clemson.edu/joe/vol47/iss1/6  36. McLeod, E., Jensen, K. L., DeLong, K. L., & Griffith, A. 2019. A Multiple Indicators, Multiple Causes Analysis of Farmers' Information Use. The Journal of Extension, 57(3), Article 20. https://doi.org/10.34068/joe.57.03.20 |
| 4 | The data used are quite old. More recent data would give more interesting results. | The following explanation was added:  <Data and descriptive statistics>  "To empirically prove the effect of the agricultural extension, the Agricultural Census 2010 microdata published by the PCBS was used. At the time this study was conducted, only the microdata of the Agricultural Census 2010 was available."  <Conclusion>  "At the time this study was conducted, only individual data from the Agricultural Census 2010 were publicly available. However, in the future, when the Agricultural Census 2020 microdata becomes public and available, updated estimates will be made using it." |
| 5 | On page 9, just after Table 2, the interpretation of the "Distance from West Bank Barrier" variable seems the opposite of the results. The author will need to revise the interpretation at this level. | The interpretation written in the original manuscript was correct. In this study, distance means remoteness from the West Bank Barrier and Israeli Settlements (Not closeness). For clear explanation, the following sentence was added after Table 2.  "In this analysis, distance means remoteness from the West Bank Barrier and Israeli Settlements." |
| 6 | In Table 3, since the author used logistic estimation, are these the marginal effects that are presented in the table? | Table 3 presents coefficient of each variable. Average marginal effects were also added to Table 3. |
| 7 | In Tables 2 and 3, the R-squares are low, which does not validate the results presented. | Since the microdata used in this study was cross-sectional data, the value of adjusted R-squared and pseudo-R-squared is not so high compared to time series analysis and panel data. The following explanation was added after Table 2 and 3:  "Since the microdata used in this study is cross-sectional data, the value of adjusted R-squared is not so high. However, the following could be interpreted from the results."  "Since the microdata used in this study is cross-sectional data, the value of pseudo-R-squared is not so high. However, the following could be interpreted from the results." |

**Reviewer #2**

| No. | Comment | Revision results and explanation |
| --- | --- | --- |
| 1 | need to provide explanation in the Abstract and somewhere under the “Introduction”, why they chose the Occupied Palestinian Territories (OPT) to conduct their study on the OPT. | The following sentence was added to Abstract and Introduction:  <Abstract>  "The fundamental objective of this study is to make recommendations for further improvement of the agricultural extension by the Palestinian Authorities in the West Bank of the Palestinian territories as an urgent issue."  <Introduction>  "The West Bank of the Palestinian territories has been under Israeli occupation since 1967. Under such an environment, Palestinian farmers continue to farm for their livelihood. The West Bank was selected as the target area for this study because assistance to such farmers was recognized as an urgent issue." |
| 2 | I recommend that this expression “the West Bank Barrier” to be replace, under the Abstract and throughout the Ms. with “the “Israeli Segregation Wall”, as the used expression in the Ms. leads to misunderstanding. It is not a “West Bank’s” but Israeli, and it is not a barrier but monstrous wall. Please see also the references suggested to support this. | UNRWA and UNOCHA under United Nations use "the West Bank Barrier" for official documents. Therefore, "the West Bank Barrier" was used in this study. |
| 3 | Using the data of the Agricultural Census 2010, the authors need to explain why they relied on data 2010, considering the fact that it is relatively old data (13 years old). | At the time this study was conducted, only the microdata of the Agricultural Census 2010 was available. A subsequent agricultural census was conducted in 2021, but no microdata has been published. The following explanation was added:  <Data and descriptive statistics>  "To empirically prove the effect of the agricultural extension, the Agricultural Census 2010 microdata published by the PCBS was used. At the time this study was conducted, only the microdata of the Agricultural Census 2010 was available."  <Conclusion>  "At the time this study was conducted, only individual data from the Agricultural Census 2010 were publicly available. However, in the future, when the Agricultural Census 2020 microdata becomes public and available, updated estimates will be made using it." |
| 4 | “… the Israeli authority ….” To be replaced throughout the Ms. with “… the Israeli occupation authorities …” | UNRWA and UNOCHA under United Nations use "the Israeli Authorities" for official documents. Therefore, "the Israeli Authorities" was used in this study. |
| 5 | Under the Abstract: “… such as improved crop varieties, chemical fertilizers, organic fertilizers, and pesticides” …. “estimated using nearest-neighbor matching method, were by 7.1, 7.7, 5.4, 6.8, and 3.8 percentage points respectively the examples provided are 4 and the results are 5, so they are not equal “respectively”. | It was modified as follows:  <Abstract>  "The results showed that agricultural extension has positively and significantly affected the adoption of five technologies, namely improved crop varieties, chemical fertilizers, organic fertilizers, pesticides, and biological control. The estimated increase in the adoption rate of those technologies as the average treatment effects on the treated (ATT) by the nearest-neighbor matching method were by 7.1, 7.7, 5.4, 6.8, and 3.8 percentage points respectively." |
| 6 | On Page 3, last two lines: “The fruit yield was about 53%, field crop yield was about 33% and olive yield was about 36% of Israel’s crop yield.” Is not clear: Is it the ““The Palestinian fruit yield was about 53%, Also, please council here and cite them some of Hilmi S. Salem’s publications, given below. | The following recommended study was cited as follows:  <Introduction-Agricultural extension under Israeli occupation>  "Constraints on the use of water resources by Israel are one reason for the shortage of irrigation water. In 2007–2008, a 35–40% reduction in the production of rainfed crops (wheat, forage, olives, grapes, and other fruit trees) was observed due the shortage of irrigation water [15].  <Reference>  15. Yihdego, Y., Salem, H.S., and Muhammed, H.H. 2019. Agricultural pest management policies during drought: Case studies in Australia and the State of Palestine. Natural Hazards Review. 2019, 20(1-February), 1–10. https://doi.org/10.1061/(ASCE)NH.1527-6996.0000312 |
| 7 | Page 5, Second Parag.: coefficients of 𝛽0, 𝛽1, … | It was modified. |
| 8 | Page 5, Third Parag.: “We used the following binary ….” “As covariates, we included distances from the West Bank Barrier and Israeli settlements …” “We also referred to earlier studies to select independent variables in this study.” It is preferred not to use we, I, ours, etc. Please use instead, throughout the Ms., the passive form, such as: the following binary …. were used, and so forth. | All the expressions were changed to passive form throughout this manuscript, and it was proofread by a native English speaker. |
| 9 | “Much of the West Bank is arid, and Israel has restricted use of water resources, which has led to chronic shortage of irrigation water [7].” This is not correct; the West Bank has the very good fertile land in Historic Palestine that includes the occupied West Bank and Israel, especially what is classified, according to Oslo, as Area C, which is totally controlled by the Israeli occupiers. | The sentence was modified as follows:  <Analytical framework>  "The Israeli Authorities have restricted its use of water resources in much of the West Bank, leading to chronic irrigation water shortages [7]. |
| 10 | Probably under “3. Analytical framework” – probably in the end of this part of the study – the authors need to clarify what kind of software used and applied for the analyses and the results obtained. Is it already available and the authors just applied it, or they had developed it for the purpose(s) of the present study. | The following sentence was added:  <Analytical framework>  "All estimates in this study were performed using the statistical analysis application Stata/IC 16.1 developed by STATA Corp LLC." |
| 11 | Under 4., 1st Paragr., 1st two lines, the authors say: “To empirically prove the effect of the agricultural extension, we used the Agricultural Census 2010 microdata published by the PCBS.” As mentioned above, the 2010 Census data is relatively old and, therefore, the authors need to provide explanation why the used such relatively old data, instead of relatively most recent data, if available. | At the time this study was conducted, only the microdata of the Agricultural Census 2010 was available. A subsequent agricultural census was conducted in 2021, but no microdata has been published. The following explanation was added:  <Data and descriptive statistics>  "To empirically prove the effect of the agricultural extension, the Agricultural Census 2010 microdata published by the PCBS was used. At the time this study was conducted, only the microdata of the Agricultural Census 2010 was available."  <Conclusions>  "At the time this study was conducted, only individual data from the Agricultural Census 2010 were publicly available. However, in the future, when the Agricultural Census 2020 microdata becomes public and available, updated estimates will be made using it." |
| 12 | Under 4. “Geographic data on the West Bank Barrier, Israeli settlements, land classification by the Oslo II Accord, and boundaries of Palestinian localities was obtained from the …” were obtained … | It was modified. |
| 13 | Page 7, the first two lines: “have different social and natural environments, we treated them separately and used only the data for the West Bank in this study.” Having different social and natural environments should make the authors to consider them. I see the opposite, meaning because they (WE & GS) have different characteristics, they should be considered for comparison purposes both in the analyses applied and the results obtained. | The following sentence was added:  <Conclusions>  "The Gaza Strip could not be included in the same model in this study because it has no Israeli settlements. In the future, it will be necessary to make similar estimates and compare them with those in the West Bank regarding the impact of the conflict and the effects of agricultural extension, taking into account the political situation and natural environment in the Gaza Strip." |
| 14 | Figures 1 and 2 (the maps) are of bad quality. They should be highly improved or replaced by others of much better quality. | These two maps were deleted due to copyright considerations. |
| 15 | Page 7, 2nd Parag., “Fig 1 shows the shortest distance from the localities to the West Bank Barrier and Israel settlements. Fig 2 shows the classification of localities according to the Oslo II Accord.” OK, fine, but what it mean within the perspectives of the Manuscript. Please explain. | The following sentence was added:  <Data and descriptive statistics>  The classification of localities based on the Oslo II Accord means the difference in authorities in charge of administration for each locality, which also affects agricultural extension. |
| 16 | Page 11 under the Table, the authors state: “As mentioned, most of the extension officers of the Palestinian authority as of 2010 were male officers (Please provide here a reference or more supporting your argument). Therefore, based on their religious background, it is inferred that male farmers, rather than female, are more likely to contact extension officers (Please provide here a reference or more supporting your argument). Regarding the holders’ educational background, the holders with secondary or associate diploma and bachelor’s degree or above are more likely to receive agricultural extension than those with preparation or less.” (Please provide here a reference or more supporting your argument). Otherwise delete these statements if cannot support them with recent published research. | The following sentence was added:  <Introduction>  " In the West Bank, extension officers in the Directorates of Agriculture are mainly in charge of the agricultural extension services. The total number of extension officers was 168, of which 123 (73.2%) were male offices and 45 (26.8%) were female officers [19]."  19. Center for Integrated Rural Development-”CIRD”. Assessment of the Public Agricultural Extension System of Palestine & Recommendations for Improvements, 2011. https://ershad.moa.pna.ps/downloads/reports/20120717112803.pdf  <Results>  "Female farmers work in farming and agro-processing on family farms and contribute significantly to the agricultural sector and GDP in the West Bank [32]."  32. Salem, Hilmi. 2019. Agriculture Status and Women’s Role in Agriculture Production and Rural Transformation in the Occupied Palestinian Territories. Journal of Agriculture and Crops. 2019, 5(8): 132-150. 5. 132-150. 10.32861/jac.5(8)132.150. https://doi.org/10.32861/jac.5(8)132.150  "Female farmers prefer female extension officers. But number of female extension officers are insufficient, thereby hindering outreach to female farmers since cultural traditions often restrict male extension agents from providing services to female farmers. Female farmers prefer female extension officers [33]."  33. Food and Agriculture Organization of the United Nations (FAO). Country Gender Assessment of the agriculture and rural sector - Palestine; 2021. https://www.fao.org/family-farming/detail/en/c/1411724/  "Regarding the holders’ educational background, the holders with secondary or associate diplomas and bachelor’s degrees or above are more likely to receive agricultural extension than those with preparation or less. Several previous studies have shown that the higher education level of farmers positively influences their use of information sources [34] [35] [36]."  34. Edge, B., Velandia, M., Lambert, D. M., Roberts, R. K., Larson, J. A., English, B. C., Boyer, C., Rejesus, R., & Mishra, A. 2017. Changes in the Use of Precision Farming Information Sources Among Cotton Farmers and Implications for Extension. The Journal of Extension, 55(2), Article 16. https://tigerprints.clemson.edu/joe/vol55/iss2/16  35. Jensen, K. L., English, B. C., & Menard, R. 2009. Livestock Farmers' Use of Animal or Herd Health Information Sources. The Journal of Extension, 47(1), Article 6. https://tigerprints.clemson.edu/joe/vol47/iss1/6  36. McLeod, E., Jensen, K. L., DeLong, K. L., & Griffith, A. 2019. A Multiple Indicators, Multiple Causes Analysis of Farmers' Information Use. The Journal of Extension, 57(3), Article 20. https://doi.org/10.34068/joe.57.03.20 |
| 17 | Page 12, last two lines: “and the location of Hebron H1”, please explain this and give a reference if possible. | The following sentence was added:  <Results-Balancing test of the PSM>  "Hebron H1 in Hebron city is an area administered by the Palestinian Authorities under the Hebron Protocol of 1997 [38]."  38. Maymind, Ilana Ph.D. 2019. "Neuman, Tamara. Settling Hebron. Jewish Fundamentalism in a Palestinian City. Philadelphia, PA: University of Pennsylvania Press, 2018.," Journal of International and Global Studies: Vol.10:No.2, Article 15. https://digitalcommons.lindenwood.edu/jigs/vol10/iss2/15 |
| 18 | Page 16, first three lines, the authors state: “These varieties are also available to farmers in the West Bank. The conflicted-affected agricultural holdings must adopt those varieties with high yields and resistance to pests and diseases to maintain their livelihoods on the limited farmlands.” Please support this statement with citations from Yihedogo et al. (2019). Agricultural pest management policies …. Please see below. “Additionally, as one of the measures of adaptation against climate change, the use of improved drought- resistant varieties could improve agricultural incomes” Please support this statement with citations from Yihedogo et al. (2019). Agricultural pest management policies …. Please see below. | The following sentences was added:  <Discussion-Improved crop varieties>  "The best approach to pest management is the use of disease and pest-resistant varieties or breeds in the West Bank. The Palestinian Authorities can financially contribute in order to help reduce the impacts of pests on drought-affected farmers [15]."  15. Yihdego, Y., Salem, H.S., and Muhammed, H.H. 2019. Agricultural pest management policies during drought: Case studies in Australia and the State of Palestine. Natural Hazards Review. 2019, 20(1-February), 1–10. https://doi.org/10.1061/(ASCE)NH.1527-6996.0000312 |
| 19 | Page 16, 2nd Parag. 3rd line: “of fertilizers in Palestinian was 120–150 NIS” in the Occupied Palestinian Territories (OPT). | It was modified. |
| 20 | Page 16, 2nd Parag. 6 and 7, the authors state: “Therefore, chemical fertilizers in the Palestinian territories were priced 1.6 to 2.0 times higher than those in Israel. Since 2008,” The authors need to present here the discrimination policies that the Israeli occupation authorities apply against the Palestinians – the Indigenous population of the land. They need to cite here Salem, H.S. (2019a). No sustainable development….. and Salem, H.S. (2020). Geopolitical challenges, complexities, and future uncertainties…. Please see the references given below. | It was already mentioned as follows in <Discussion-Fertilizer>.  "Since 2008, the Israeli Authorities have restricted the import of items that could be diverted for military purposes (dual-use goods) into the West Bank and Gaza Strip [45]. The regulatory list also includes the following chemical fertilizers used extensively worldwide [45]: ammonium nitrate, potassium nitrate, urea, urea nitrate, NPK 17-10-27 compound fertilizer, NPK 20-20-20 compound fertilizer, etc. Therefore, few varieties of chemical fertilizers are available in the Palestinian territories, which limits farmers’ options." |
| 21 | Page 16, under Parag. 6.4. Pesticides: The authors need to support their arguments with recently published research, locally or internationally. | The following sentence was added:  <Discussion-Pesticides>  "It is known that several pesticides that are internationally banned or have expired are imported to the West Bank [48] and nearly 50% of the pesticides used in the Palestinian territories were internationally illegal [49]. Furthermore, the labels of pesticides imported into the West Bank do not contain chemical specifications or safety instructions written in Arabic. Most Palestinian farmers cannot understand and use it safely [48]."  48. Salem, H.S. 2019. No sustainable development in the lack of environmental justice. Environmental Justice, 12(3-June):140–157. https://doi.org/10.1089/env.2018.0040 https://www.liebertpub.com/doi/10.1089/env.2018.0040  49. Watts, M, Lee T, and Aidy H. Pesticides and Agroecology in the Occupied West Bank. Conclusions from a Joint APN-PANAP Mission in Palestine, May 2016; 2017. https://panap.net/resource/pesticides-and-agroecology-in-the-occupied-west-bank/ |
| 22 | Page 17, under “Biological Control”, please council the publication of “Yihdego, Y., Salem, H.S., and Muhammed, H.H. (2019). Agricultural pest management policies during drought: …..” and cite there. | Since this reference is not directly related to this study, it was added. |
| 23 | Under the “Conclusion”, do NOT refer to any references cited previously in the text of the manuscript, but use ONLY your conclusions. | It was modified. No citations are included, and all are our own conclusions. |
| 24 | Under “Acknowledgments”, please consider adding the following: The Authors express their thanks to Prof. Dr. Hilmi S. Salem, Sustainable Development Research Institute, Occupied Palestine, for his valuable inputs, improvements, and suggestions, as well as his critical review of the manuscript. Also, please correct this: The author thanks The authors thank ….. | We thank Mr. Salem for his detailed review. However, from the point of view of anonymity in review process, we would like to refrain from including his name in the acknowledgment. Therefore, the following sentences was added:  <Acknowledgment>  "The authors would like to thank two anonymous reviewers who provided invaluable feedback on the structure and content of this manuscript, and the PCBS for providing microdata of the Agricultural Census 2010." |
| 25 | Please council the references given below, cite them and include them under “References” given in the manuscript. | As noted above, some of the recommended references that are directly relevant to this study were added. |
| 26 | Please cite the following references and add them to the List of References in the end of the Ms. where are being best appropriate and fitting. They are authored by the Reviewer (Prof. Hilmi S. Salem; a Palestinian scholar and citizen) and they describe well the status of land, water, agriculture, food security, socioeconomics, geopolitics, political conflict, gender, and so forth of the study areas investigated by the Authors of the Ms. Please the most recent publication highlighted in yellow and given in the end of the List below. These publications can be easily found and downloaded using the Links provided. The most recent publication by the Reviewer is the first one highlighted in yellow. | As noted above, some of the recommended references that are directly relevant to this study were added. |

**Request from the Editorial Staff**

| No. | Comment | Revision results and explanation |
| --- | --- | --- |
|  | Please ensure that all your territorial descriptions in the manuscript adhere to our submission guidelines (https://journals.plos.org/plosone/s/best-practices-in-research-reporting). Territorial descriptions in submitted manuscripts should follow international treaties and conventions. PLOS remains neutral on any jurisdictional claims expressed or implied in published manuscript texts, maps and institutional affiliations. | For words related to Palestine and Israel, the words used in official documents of the United Nations, an international organization were selected in this manuscript. |
|  | In addition, we noticed that Reviewer#2 has recommended a number of citations as a part of their review. We would recommend that you thoroughly evaluate these requested references and determine whether the articles are relevant to the current study. You may feel free to disregard references without tangible relevance to the study reported in the manuscript. | As noted above, some of the recommended references that are directly relevant to this study were added. |
|  | 1. Please ensure that your manuscript meets PLOS ONE's style requirements, including those for file naming. | It was confirmed and the manuscript was modified following the PLOS ONE style. |
|  | 2. Please ensure that all your territorial descriptions in the manuscript adhere to our submission guidelines on territorial descriptions (https://journals.plos.org/plosone/s/best-practices-in-research-reporting)  Territorial descriptions  Territorial descriptions in submitted manuscripts should follow international treaties and conventions. PLOS remains neutral on any jurisdictional claims expressed or implied in published manuscript texts, maps and institutional affiliations. | For words related to Palestine and Israel, the words used in official documents of the United Nations, an international organization were selected in this manuscript. |
|  | 3. We note that Figures 1 and 2 in your submission contain [map/satellite] images which may be copyrighted. All PLOS content is published under the Creative Commons Attribution License (CC BY 4.0), which means that the manuscript, images, and Supporting Information files will be freely available online, and any third party is permitted to access, download, copy, distribute, and use these materials in any way, even commercially, with proper attribution. For these reasons, we cannot publish previously copyrighted maps or satellite images created using proprietary data, such as Google software (Google Maps, Street View, and Earth). For more information, see our copyright guidelines: http://journals.plos.org/plosone/s/licenses-and-copyright. | These two maps were deleted to avoid copyright issues. |
