# Supplementary material for: Effect of agricultural extension on technology adoption by Palestinian farmers under Israeli occupation in the West Bank
Source: PLoS One. 2023 Nov 8;18(11):e0294023. doi: 10.1371/journal.pone.0294023 (PMC10631675; doi:10.1371/journal.pone.0294023)
Supplement: Attachment — Submitted filename: Response to Reviewers PLOS ONE Nakamura 230928.docx [file pone.0294023.s004.docx]

**Response to reviewers**

| No. | Comments | Revision results and explanation |
| --- | --- | --- |
| 1 | Abstract: “Occupied Palestinian Territories (OPT) as an urgent issue.” | Modified. |
| 2 | Abstract and throughout the paper: “by the Israeli Occupation Authorities …” | Modified throughout the manuscript. |
| 3 | Throughout the paper, the authors still use the term “West Bank Barrier,” though I asked them to replace it with the term “Israeli Segregation Wall,” as widely used by local and international researchers and documented in a plenty of literature. It is not a small barrier or fence between good neighbors but a giant monstrous wall intended to confiscate huge Palestinian lands and water resources behind it, and to significantly and negatively impact the life of the entire Palestinian population in the occupied West Bank, including occupied East Jerusalem. Its length has reached, so far, about 800 km surrounding the occupied West Bank. Please see this reference of mine, in which you find some description of the Israeli Segregation Wall built illegally on Palestinian lands of the occupied West Bank. Please feel free to cite and reference it in your current Ms. and other future works:  Salem, H.S. (2011). Social, environmental and security impacts of climate change on the Eastern Mediterranean. In: Brauch, H.G., Spring, Ú.O., Mesjasz, C., Grin, J., Kameri-Mbote, P., Chourou, B., Dunay, P., Birkmann, J. (Eds.): Coping with Global Environmental Change, Disasters and Security – Threats, Challenges, Vulnerabilities and Risks. The Institute for Environment and Human Security, United Nations University (UNU-EHS). Hexagon Series on Human and Environmental Security and Peace, Berlin – Heidelberg – New York: Springer-Verlag. PP: 421–445. 1815pp. Available at:  <https://link.springer.com/chapter/10.1007/978-3-642-17776-7_23> [https://www.researchgate.net/publication/299562984_Social_Environmental_and_Security_Impact](https://www.researchgate.net/publication/299562984_Social_Environmental_and_Security_Impacts_of_Climate_Change_of_the_Eastern_Mediterranean) [s_of_Climate_Change_of_the_Eastern_Mediterranean](https://www.researchgate.net/publication/299562984_Social_Environmental_and_Security_Impacts_of_Climate_Change_of_the_Eastern_Mediterranean) | Modified throughout the manuscript. Thank you for recommending the informative paper. However, we thought that the other papers you recommended were more appropriate, so we cited the following three papers in the sentences explaining the effects of the wall and the settlements.  <P.4>  "The wall grabs fertile agricultural land, isolates Palestinian communities in enclaves undermining the territorial contiguity between Palestinian villages, and limits access to natural resources causing water shortages [13, 14]."  Reference:  *13. Salem, H.S. 2020. Social, Environmental and Security Impacts of Climate Change on the Eastern Mediterranean. https://doi.10.1007/978-3-642-17776-7_239*  *14. Salem, H.S. 2023. Potential Solutions for the Water Conflict between Palestinians and Israelis. A Book Chapter (PP: 123–185) In: Hussein A. Amery (Ed.): “Enhancing Water Security in the Middle East”. MENA Water Security Task Force, Al-Sharq Strategic Research, Al-Sharq Forum, Istanbul, Turkey; and Colorado School of Mines, Denver, CO, USA.*  <P.4>  "These settlements discharge wastewater, affecting surrounding farmlands and irrigation water [17]."  Reference:  *17. Salem, H.S. 2023. Socioeconomic, environmental, and health impacts of reusing treated wastewater in agriculture in some Arab countries, including occupied Palestine, in view of climate change. Natural Resources Conservation and Research, Volume 6, Issue 2. 22 Pages. “A special Issue: Climate-Adaptive Approaches to Risk Monitoring and Sustainable Water Resources Management." (Published online: 29 August 2023), EnPress Publisher, El Monte, CA, USA.* |
| 4 | Abstract and in other locations: Palestinian Authorit~~ies~~ Authority | Modified throughout the manuscript. |
| 5 | Abstract: Using the data of the Agricultural Census 2010, The authors did not explain enough why they used this relatively old data!!!! | Aggregated data from the Agricultural Census 2021 has been made public, but microdata on individual farmers has not been made public, and it is unclear whether it will be made public in the future. Therefore, microdata from the agricultural census 2010 was used for the empirical analysis of this study. I would like to use the micro data from the Agricultural Census 2021 if they were officially available, but unfortunately, it is not available even now. We hope for your understanding. Therefore, the manuscript was modified as follows.  <P.1>  "The data from the Agricultural Census 2010, which was the only microdata officially published and available at the time of this study, was used. Then, the Propensity Score Matching (PSM) method was employed to mitigate the endogenous bias caused by self-selection by farmers in receiving the agricultural extension." |
| 6 | Under Introduction: before and after the 2014 ~~Gaza conflict~~ Israeli war on the Gaza Strip … | Modified. |
| 7 | Page 6: The Israeli Authorities have restricted its use of water resources in much of the West Bank, leading to chronic irrigation water shortages[7]. Please consider adding here (in addition to the 7^th^ reference) this up-to-date (March 2023) reference which was also recommended in the first review, and also the following one which has been published just today. It is belied that both reference are relevant to the Ms.:  Salem, H.S. (2023). Potential Solutions for the Water Conflict between Palestinians and Israelis. A Book Chapter (PP: 123–185) In: Hussein A. Amery (Ed.): “Enhancing Water Security in the Middle East”. MENA Water Security Task Force, Al-Sharq Strategic Research, Al-Sharq Forum, Istanbul, Turkey; and Colorado School of Mines, Denver, CO, USA. Published on 20 March 2023. <https://research.sharqforum.org/mena-water-security-task-force/> [https://www.researchgate.net/publication/367190828_Chapter_4_Potential_Solutions_for_the_Wa](https://www.researchgate.net/publication/367190828_Chapter_4_Potential_Solutions_for_the_Water_Conflict_Between_Palestinians_and_Israelis) [ter_Conflict_Between_Palestinians_and_Israelis](https://www.researchgate.net/publication/367190828_Chapter_4_Potential_Solutions_for_the_Water_Conflict_Between_Palestinians_and_Israelis)  Salem, H.S. (2023). Socioeconomic, environmental, and health impacts of reusing treated wastewater in agriculture in some Arab countries, including occupied Palestine, in view of climate change. Natural Resources Conservation and Research, Volume 6, Issue 2. 22 Pages. “A special Issue: Climate- Adaptive Approaches to Risk Monitoring and Sustainable Water Resources Management." (Published online: 29 August 2023), EnPress Publisher, El Monte, CA, USA. DOI: <http://dx.doi.org/10.24294/nrcr.v6i2.2229>  <https://systems.enpress-publisher.com/index.php/NRCR/article/view/2229> [https://www.researchgate.net/publication/372317053_Socioeconomic_environmental_and_health_](https://www.researchgate.net/publication/372317053_Socioeconomic_environmental_and_health_impacts_of_reusing_treated_wastewater_in_agriculture_in_some_Arab_countries_including_occupied_Palestine_in_view_of_climate_change) [impacts_of_reusing_treated_wastewater_in_agriculture_in_some_Arab_countries_including_occupi](https://www.researchgate.net/publication/372317053_Socioeconomic_environmental_and_health_impacts_of_reusing_treated_wastewater_in_agriculture_in_some_Arab_countries_including_occupied_Palestine_in_view_of_climate_change) [ed_Palestine_in_view_of_climate_change](https://www.researchgate.net/publication/372317053_Socioeconomic_environmental_and_health_impacts_of_reusing_treated_wastewater_in_agriculture_in_some_Arab_countries_including_occupied_Palestine_in_view_of_climate_change) | Thank you for recommending the informative latest papers. the three papers were cited in relevant sentence explaining the situation in Palestine as mentioned above.  <P.4>  "The wall grabs fertile agricultural land, isolates Palestinian communities in enclaves undermining the territorial contiguity between Palestinian villages, and limits access to natural resources causing water shortages [13, 14]."  Reference:  *13. Salem, H.S. 2020. Social, Environmental and Security Impacts of Climate Change on the Eastern Mediterranean. https://doi.10.1007/978-3-642-17776-7_239*  *14. Salem, H.S. 2023. Potential Solutions for the Water Conflict between Palestinians and Israelis. A Book Chapter (PP: 123–185) In: Hussein A. Amery (Ed.): “Enhancing Water Security in the Middle East”. MENA Water Security Task Force, Al-Sharq Strategic Research, Al-Sharq Forum, Istanbul, Turkey; and Colorado School of Mines, Denver, CO, USA.*  <P.4>  "These settlements discharge wastewater, affecting surrounding farmlands and irrigation water [17]."  Reference:  *17. Salem, H.S. 2023. Socioeconomic, environmental, and health impacts of reusing treated wastewater in agriculture in some Arab countries, including occupied Palestine, in view of climate change. Natural Resources Conservation and Research, Volume 6, Issue 2. 22 Pages. “A special Issue: Climate-Adaptive Approaches to Risk Monitoring and Sustainable Water Resources Management." (Published online: 29 August 2023), EnPress Publisher, El Monte, CA, USA.* |
| 8 | Page 18: (New Israel Shekel) …. | Modified. |
| 9 | Under “Conclusions”: However, the effects of the conflict are diverse and devastating on Palestinian farmers in the Occupied Palestinian Territories (West Bank and Gaza Strip). They, however, ~~and~~ cannot be fully explained by the variables used in this study. Therefore, it is still necessary to find and use variables that more accurately represent the impact of the conflict. | Modified. |
| 10 | In the West Bank, the Israeli Occupation Authorities …. | Modified. |
| 11 | The Gaza Strip could not be included in the same model in this study because it has no Israeli settlements. Yes, but the Gaza Strip is totally besieged and blockaded by the Israeli Occupation Authorities since 2006. Please mention this note in your Ms. and support it with references. | As you pointed out, it is clear that the Gaza fence have a major impact on agriculture in the Gaza Strip. However, the impact of Israeli occupation is different in the West Bank and Gaza Strip. For example, the destruction of farmland due to air strikes is a particularly large factor influencing farmers' behavior. Therefore, we recognize the need to establish a different model with different variables used for empirical analysis. In this study, the only data available was location information on the Segregation Wall and settlements in the West Bank, so this study focused only on the West Bank. If this paper is accepted and we can get the location information of the airstrike, we would like to use the similar method and establish a new model specific to the Gaza Strip. We hope for your understanding. Based on your advice, I added the following sentences to the manuscript.  <P.8>  "The impacts of the conflict on agriculture in the Gaza Strip are different from those of the West Bank: repeated airstrikes, blockades causing the movement restriction of people and goods, less accessibility of agricultural inputs, and restricted access to external markets [35, 36]. Therefore, it was determined that it was necessary to establish a specific model for the Gaza Strip different from the West Bank, which includes specific variables reflecting properly these particular factors, and the Gaza Strip could not be included in this study."  Reference:  *35. Jacques M., Ahmad U. and Jean Michel S. (2019). Study on Small-scale Agriculture in the Palestinian Territories. Centre de coopération internationale en recherche agronomique pour le développement (CIRAD).*  *36. United Nations Conference on Trade and Development (UNCTAD). The Economic Costs of the Israeli Occupation for the Palestinian People: The Impoverishment of Gaza under Blockade; 2020.* |
| 12 | References: 32. Salem, H.S. 2019. Agriculture Status … | Modified. |
| 13 | References: 48. Salem, H.S. 2019. No sustainable development …. | Modified. |
