# Supplementary material for: Effect of agricultural extension on technology adoption by Palestinian farmers under Israeli occupation in the West Bank
Source: PLoS One. 2023 Nov 8;18(11):e0294023. doi: 10.1371/journal.pone.0294023 (PMC10631675; doi:10.1371/journal.pone.0294023)
Supplement: Attachment — Submitted filename: Response to Editor Comments PLOS ONE Nakamura 231014.docx [file pone.0294023.s005.docx]

**Response to** **Additional Editor Comments**

| No. | Comments | Revision results and explanation |
| --- | --- | --- |
| 1. | The abstract needs to be reviewed. There is no need for the first sentence. It can be deleted. Please give a brief description of the problem statement following the objectives, the methodology, and data used in the paper. Then talk about the results and their implication as policy recommendations. | The abstract was reviewed as advised. The first sentence was deleted, and the background was simplified. The objective was clarified following results and their implication as policy recommendations. |
| 2. | The following papers can be helpful  Ali, E. (2021). Farm households’ decision of adoption of climate-smart practices in subsistence agriculture: Evidence from Northern Togo. Environmental Management, 67: 949–962 https://doi.org/10.1007/s00267-01436-3  Ali, E., Mounkounti, Y. (2020). Adoption de la technologie Biofeed dans la lutte contre la mouche des fruits au Togo. Sytèmes Alimentaires/ Food Systems, 5(1), 157-180. https://dx.doi.org/10.15122/isbn.978-2-406-11062-0.p.0157  Ali., E., Awade, E.N., Abdoulaye, T. (2020). Gender and impact of climate change adaptation on soybean farmers’ revenue in rural Togo, West Africa. Cogent Food & Agriculture 6(1): 1743625. https://doi.org/10.1080/23322039.2020.1783910 | Thank you for recommending the informative papers.  The first paper has already been cited. A third paper related to this study was cited and used as the basis for variable selection. |
|  |  |  |
|  |  |  |
